# Supplementary material for: Causal Relationship between Aging and Anorexia Nervosa: A White-Matter-Microstructure-Mediated Mendelian Randomization Analysis
Source: Biomedicines. 2024 Aug 16;12(8):1874. doi: 10.3390/biomedicines12081874 (PMC11351342; doi:10.3390/biomedicines12081874)
Supplement: Supplementary file 1 [file biomedicines-12-01874-s001.zip › Supplementary Figure/Figure S7/Single SNP plot of MR analysis for ubm-b-1972 to AN.pdf]

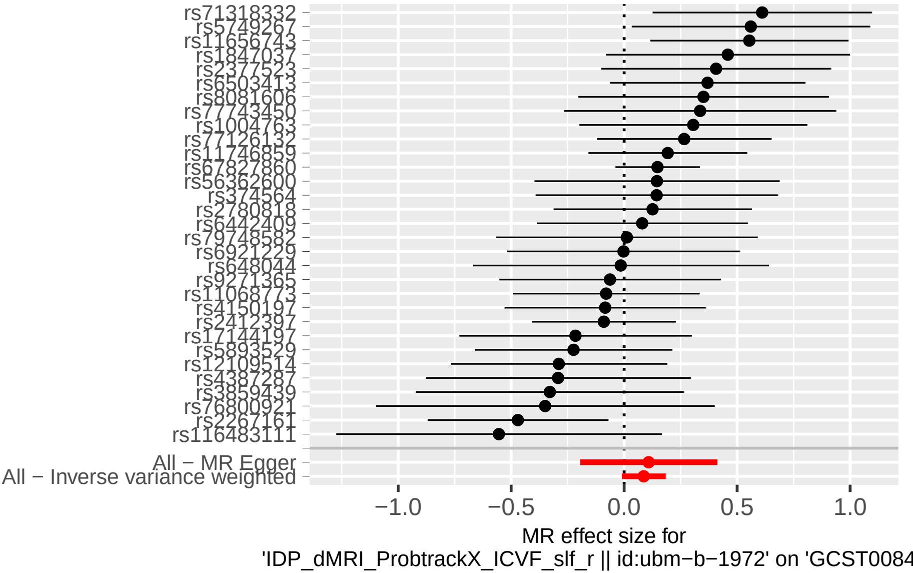
rs71318332
rs5749267
rs11656743
rs1847037
rs2377523
rs6503413
rs8081606
rs77743450
rs1004763
rs77126132
rs11746859
rs67827860
rs56362600
rs374564
rs2780818
rs6442409
rs79748582
rs6921229
rs648044
rs9271365
rs11068773
rs4150197
rs2412397
rs17144197
rs5893529
rs12109514
rs4387287
rs3859439
rs76800921
rs2267161
rs116483111
All − MR Egger
All − Inverse variance weighted
−1.0
−0.5
0.0
0.5
1.0
MR effect size for
'IDP_dMRI_ProbtrackX_ICVF_slf_r || id:ubm−b−1972' on 'GCST0084
